# Monitoring Antimicrobial Susceptibility of *Neisseria gonorrhoeae* Isolated from Bangladesh during 1997-2006: Emergence and Pattern of Drug-resistant Isolates

**DOI:** 10.3329/jhpn.v28i5.6152

**Published:** 2010-10

**Authors:** Monir Uddin Ahmed, Faisal Arif Hasan Chawdhury, Maqsud Hossain, Syed Zafar Sultan, Mansur Alam, Gazi Salahuddin, Ashraful Alam, Khairun Nessa, Shamsun Nahar, Shama-A-Waris, Anadil Alam, Motiur Rahman

**Affiliations:** ^1^ Laboratory Sciences Division, ICDDR,B, GPO Box 128, Dhaka 1000, Bangladesh; ^2^ Institute of Food and Radiation Biology, Bangladesh Atomic Energy Commission, GPO Box 3787, Dhaka 1000, Bangladesh; ^3^ Department of Medical Microbiology, University of Manitoba, Winnipeg, MB, Canada; ^4^ Department of Life Sciences, North South University, Dhaka, Bangladesh; ^5^ Department of Microbiology, Biochemistry and Immunology, Morehouse School of Medicine, Atlanta, GA 30310, USA; ^6^ M.A.G. Osmani Medical College, Sylhet; ^7^ Laboratory Sciences Division, Family Health International, Asia Pacific Regional Office, Bangkok, Thailand

**Keywords:** Drug resistance, Microbial, Gonorrhoea, *Neisseria gonorrhoeae*, Sexually transmitted infections, Surveillance, Bangladesh

## Abstract

Gonorrhoea is one of the most common sexually transmitted infections (STIs) in developing countries, and the emergence of resistance to antimicrobial agents in *Neisseria gonorrhoeae* is a major obstacle in the control of gonorrhoea. Periodical monitoring of antimicrobial susceptibility of *N. gonorrhoeae* is essential for the early detection of emergence of drug resistance. In total, 1,767 gonococcal strains isolated from males and females (general population and those with high-risk behaviour) from different parts of Bangladesh were studied during 1997-2006. Minimum inhibitory concentrations of penicillin, tetracycline, ciprofloxacin, ceftriaxone, spectinomycin, and azithromycin for the isolates were determined by the agar dilution method. Isolates resistant to three or more antimicrobial agents are considered multidrug-resistant. The prevalence of plasmid-mediated penicillinase-producing *N. gonorrhoeae* (PPNG) and plasmid-mediated tetracycline-resistant *N. gonorrhoeae* (TRNG) was determined. Nine percent of the isolates were resistant to ciprofloxacin in 1997 compared to 87% in 2006. Multidrug-resistant *N. gonorrhoeae* have emerged in 1997, and 44% of the strains (n=66) isolated during 2006 were multidrug-resistant. Forty-two percent of the isolates in 2006 were both PPNG- and TRNG-positive compared to none in 1997. The rapidly-changing pattern of gonococcal antimicrobial susceptibility warrants the need for an antimicrobial susceptibility-monitoring programme, and periodical analysis and dissemination of susceptibility data are essential to guide clinicians and for successful STI/HIV intervention programmes.

## INTRODUCTION

Gonorrhoea is one of the classical sexually transmitted infections (STIs) with humans as the only host for the causative agent *Neisseria gonorrhoeae*. Despite a sharp decline in the incidence of gonococcal infection in developed countries during the last decade, gonorrhoea still remains one of the most common STIs in developing countries and a global health problem (1). The prevalence of gonococcal infection varies greatly among countries in the developed and developing world, the highest being in South and Southeast Asia, followed by sub- Saharan Africa and Latin America, where it continues to be a major public-health problem. According to the World Health Organization (WHO), there are approximately 62 million new cases of gonorrhoea worldwide annually, and almost half of the cases are estimated to occur in Southeast Asia, including Bangladesh (2).

In the absence of any effective vaccine against *N. gonorrhoeae*, control of gonococcal infection mainly depends on the identification and treatment of infected individuals. An early and successful antibiotic treatment of gonococcal infection is important for cure of the individual patient, prevention of complications, and reduction of transmission (3). Strategies for the control of gonorrhoea have relied on the use of highly-effective and often single-dose therapy administered at the time of diagnosis (4). Information on antimicrobial susceptibility of *N. gonorrhoeae* is, therefore, important for the selection of an appropriate antimicrobial agent (5).

The versatile nature of the gonococcus and its capacity to cope with changing conditions in micro-environment is a major challenge in the prevention and control of gonococcal infection (6). The continuous emergence of resistance to antimicrobial agents has made the treatment of gonorrhoea expensive, prolonged, and unpredictable. The organism acquires resistance by spontaneous mutation or by acquisition of new DNA via conjugation or transformation, and resistance may, thus, be chromosomal or plasmid-mediated (7). A single organism can have both the mechanisms of resistance, and resistance to multiple antibiotics is often common (8,9).

The ability of *N. gonorrhoeae* to become resistant to cheap and effective antimicrobial agent is well- recognized since the introduction of sulphonamide. Due to change in gonococcal antimicrobial susceptibility pattern and the emergence of penicillinase-producing *N. gonorrhoeae* (PPNG), plasmid-mediated tetracycline-resistant *N. gonorrhoeae* (TRNG) and chromosomally-mediated resistance to penicillin and tetracycline in *N. gonorrhoeae* (CMRNG^PT^), and continuing development of fluoroquinolone resistance, the Centers for Disease Control and Prevention (CDC), Atlanta, GA, USA, recommends third-generation cephalosporins as the first-line therapy for uncomplicated gonorrhoea (10).

Antimicrobial resistance in gonococci often spreads rapidly among countries, and infected travellers often appear for treatment in countries distant from the source of infection (11). Hence, data on local and regional antimicrobial resistance are important for the management and treatment of gonorrhoea.

In Bangladesh, the national STI-management guideline recommended the use of ciprofloxacin as the first-line therapy for the treatment of uncomplicated gonococcal infection during 1997-2006, and there was no systematic antimicrobial susceptibility surveillance for *N. gonorrhoeae* in the country*.* International Centre for Diarrhoeal Disease Research, Bangladesh (ICDDR,B) had initiated the antimicrobial susceptibility monitoring for *N. gonorrhoeae* in Dhaka, the capital of Bangladesh, since 1997, and it was subsequently extended to three major cities (Chittagong, Jessore, and Sylhet). We report the antimicrobial susceptibility of *N. gonorrhoeae* in Bangladesh during 1997-2006 and, in particular, the emergence and spread of multidrug-resistant *N. gonorrhoeae* in Bangladesh.

## MATERIALS AND METHODS

### Bacterial strain

All *N. gonorrhoeae* strains isolated from both male and female subjects from Dhaka and six major cities (Chittagong, Sylhet, Faridpur, Mymensingh, Barisal, and Jessore) of Bangladesh during 1997-2006 were studied. Isolates were collected as part of STI service-delivery programmes (for female sex workers in Dhaka and Jessore and for male having sex with male and male STI patients in Chittagong and Sylhet) established by ICDDR,B during 1997-2003 or as part of different STI epidemiological studies conducted during 1997-2006 in Dhaka, Jessore, Faridpur, Sylhet, Mymensingh, Chittagong, and Barisal. Isolates of *N. gonorrhoeae* were grown on modified Thayer-Martin medium (MTM) and incubated overnight at 37°C with 5-10% CO_2_. All the isolates were identified as *N. gonorrhoeae* using conventional procedures, including colony morphology, Gram-staining, oxidase and catalase tests, and carbohydrate-use test. Isolates were stored at -86°C in tryptic soy-broth (TSB) with 20% glycerol until further study.

### Minimum inhibitory concentrations

Minimum inhibitory concentrations (MICs) to penicillin, tetracycline, ciprofloxacin, ceftriaxone, spectinomycin, and azithromycin were determined by an agar dilution method described earlier (12,13). The breakpoint criteria defined by the National Committee for Clinical Laboratory Standards (NCCLS) were used for penicillin, tetracycline, ciprofloxacin, ceftriaxone, and spectinomycin, and the breakpoint criteria used for azithromycin were MIC of ≤0.25 μg/mL for susceptible, ≥0.5 of μg/mL for reduced susceptible, and ≥1 of μg/mL for resistance. Two-fold serial dilutions of antibiotics were used: penicillin (Sigma, St. Louis, MO) 0.06-32 μg/mL, tetracycline (Sigma, St. Louis, MO) 0.25-64 μg/mL, ciprofloxacin (Bayer, Hampshire, United Kingdom) 0.004-32.0 μg/mL, ceftriaxone (Sigma) 0.004-1.0 μg/mL, spectinomycin (Upjohn, Puurs, Belgium) 8.0-128 μg/mL, and azithromycin (Pfizer Inc., Connecticus, USA) 0.008-1 μg/mL. Briefly, confluent overnight culture of *N. gonorrhoeae* was suspended in TSB and adjusted to McFarland turbidity 0.5. Ten microlitre of the bacterial suspension (1×104 cfu) was spotted on GC agar (Oxoid Ltd, Basingstoke, Hampshaire, UK) plates as recommended by the WHO, containing two-fold serial dilution of antimicrobial agents using a multi-point inoculator (Mast Diagnostic Ltd., SCAN 114). The quality control of the MIC test was ensured by including seven *N. gonorrhoeae* reference strains WHO A, B, C, D, E, G, and H with known MICs in each test, by repeating each test three times and by participating in the WPRO/SEARO GASP (Western Pacific Region Office/South-East Asia Regional Office/Gonococcal Antimicrobial Surveillance Programme) external quality-assurance programme (our data were in agreement with expected results for all antimicrobial agents during the study period).

### Plasmid typing for PPNG and TRNG isolates

The plasmid type for the PPNG isolates was determined by PCR detection of β-lactamase-producing plasmid as described earlier (14), and the plasmid type for the TRNG isolates was determined by PCR amplification of *tet*M gene in 25.2-MDa conjugative plasmid (15). The primers for PPNG were designed to identify and distinguish Asia (7426)-, Africa (5599)-, and Toronto (5154)-type plasmid generating 4.9 kb, 3.1 kb, and 2.6 kb amplicons respectively. The primers for TRNG were designed to identify and distinguish American and Dutch-type plasmids generating amplicon of 1600 bp and 700 bp respectively. PCR was carried out with boiled whole-cell suspension (1×10^8^. CFU/mL) of *N. gonorr hoeae* grown overnight on MTM plate (14,15).

### Phenotypic characterization

The criteria used for phenotypic characterization of *N. gonorrhoeae* were based on plasmid- and chromosomally-mediated resistance to penicillin and tetracycline as described earlier (16). Isolates resistant to three or more antimicrobial agents were considered multidrug-resistant *N. gonorrhoeae.*

## RESULTS

In total, 1,767 gonococcal isolates were collected from males and females (population with high-risk behaviour and general population) from different parts of the country during 1997-2006. In 1997, the antimicrobial susceptibility-monitoring programme for *N. gonorrhoeae* was introduced in Dhaka, the capital of Bangladesh, and was later (1999–2003) extended to three major cities (Chittagong, Jessore, and Sylhet), in southeast, southwest and northeast parts of Bangladesh respectively. The programme was part of the STI service-delivery programme established by ICDDR,B. During 2004-2006, the programme was implemented only in Dhaka. Besides this, *N. gonorrhoeae* isolates from different epidemiological studies conducted during 1997-2006 in Dhaka, Jessore, Faridpur, Mymensingh, and Barisal were also included in the study. The number, source, and year of isolation are shown in Table 1.

**Table 1.** Year and source of *Neisseria gonorrhoeae* strains isolated from different population groups in different parts of Bangladesh during 1997-2006

Year	Total isolates (n=1,767)	No of isolates from different population groups						
		FSW (n=982)	BBSW (n=103)	HBSW (n=225)	MSM (n=30)	MT (n=81	MSTI (n=270)	FSTI (n=76)
1997	131	94^1^	30^1^^,^^7^	0	0	0	0	7^1^
1998	65	65^1^	0	0	0	0	0	0
1999	131	102^1^	29^7^	0	0	0	0	0
2000	210	208^1^	0	0	0	0	0	2^1^
2001	147	133^1^	0	0	5^2^	8^1^	1^1^	0
2002	476	66^1^	22^4^	148^1^	15^1^^,^^2^^,^^3^	34^1^	153^1^^,^^2^^,^^3^^,^^4^	38^1^^,^^2^^,^^3^
2003	340	124^1^	22^5^	0	10^1^^,^^2^^,^^3^	39^1^	116^1^^,^^2^^,^^3^^,^^4^	29^2^^,^^3^
2004	90	90^1^	0	0	0	0	0	0
2005	111	52^1^	59^5^^,^^6^	0	0	0	0	0
2006	66	48^1^	18^5^					

BBSW=Brothel-based female sex worker;

FSTI=Female STI patients;

FSW=Street-based female sex workers;

HBSW=Hotel-based sex workers;

MSM=Male having sex with male;

MSTI=Male STI patients;

MT=Male truckers;

STI=Sexually transmitted infection;

Isolates from ^1^Dhaka,

^2^Chittagong,

^3^Sylhet,

^4^Jessore,

^5^Faridpur,

^6^Barisal,

^7^Mymensingh

**Fig. 1.**
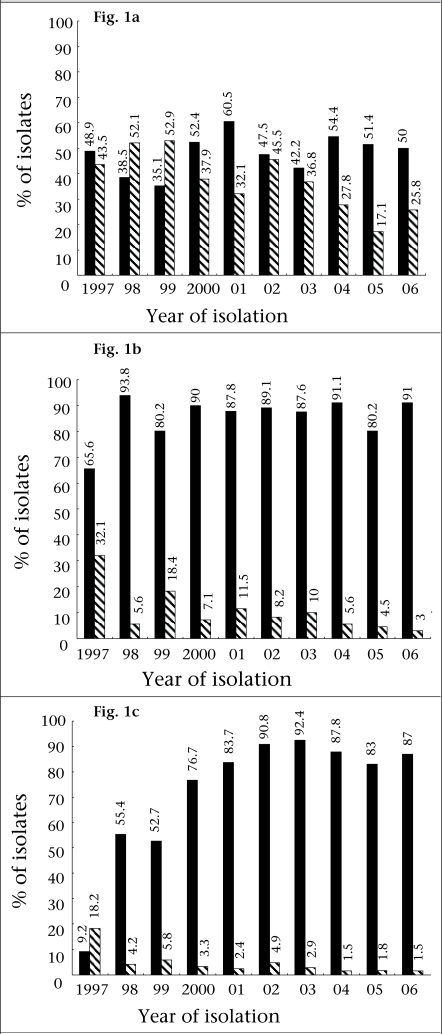
The prevalence of antimicrobial susceptibility (resistance□ and reduced susceptible▪) to penicillin, tetracycline, and ciprofloxacin of *Neisseria gonorrhoeae* isolates from Bangladesh during 1997-2006

The MICs of penicillin, tetracycline, ciprofloxacin, ceftriaxone, spectinomycin, and azithromycin for all the isolates were determined. The antimicrobial susceptibility (resistant and reduced susceptible) of the isolates to penicillin, tetracycline, and ciprofloxacin during 1997-2006 are shown in Fig. 1 (a, b, and c). Approximately 9% of the isolates in 1997 were resistant to ciprofloxacin compared to 87% in 2006 with the highest (92%) resistance in 2003. All the isolates were susceptible to ceftriaxone, azithromycin (MIC of ≤1 μg/mL), and spectinomycin, except that one isolate (0.2%) in 2002 and one each isolate in 2003, 2005, and 2006 were resistant to azithromycin and spectinomycin respectively. The MIC at which 50% and 90% of the isolates were inhibited (MIC50 and MIC90 respectively) was determined for each year (Table 2). Although most isolates in the present study were susceptible to azithromycin, a gradual increase in MIC of azithromycin was observed during 2003-2006 (Table 2). While none of the isolates had an MIC of ≥0.25 μg/mL in 1997, approximately 25% of the isolates from 2003 had an MIC of ≥0.25 μg/mL for azithromycin. No significant difference in resistance was observed among isolates collected from different populations and cities in a given year.

The MIC was determined by agar dilution method, and the breakpoint criteria used for assessing the susceptibility were those recommended by the NCCLS; Fig. 1a. Penicillin; Fig. 1b. Tetracycline; and Fig. 1c. Ciprofloxacin; MIC=Minimum inhibitory concentration; NCCLS=National Committee for Clinical Laboratory Standards

**Table 2.** MIC_50_ and MIC_90_ of 1,767 gonococcal strains isolated during 1997-2006 from males and females from general population and high-risk behaviour in Bangladesh

Year	Pen		Tet		Cip		Ceft		AZM		SPT	
	MIC_50_ μg/mL	MIC_90_ μg/mL	MIC_50_ μg/mL	MIC_90_ μg/mL	MIC_50_ μg/mL	MIC_90_ μg/mL	MIC_50_ μg/mL	MIC_90_ μg/mL	MIC_50_ μg/mL	MIC_90_ μg/mL	MIC_50_ μg/mL	MIC_90_ μg/mL
1997	1	16	2	16	0.06	1	0.015	0.06	0.06	0.12	16	32
1998	1	32	8	32	1	32	0.008	0.06	0.06	0.12	16	32
1999	1	16	4	32	1	8	0.008	0.03	0.06	0.12	16	32
2000	2	8	16	32	4	8	0.008	0.03	0.06	0.12	32	32
2001	2	8	16	64	4	8	0.004	0.015	0.06	0.12	16	32
2002	1	8	16	64	4	8	0.008	0.06	0.06	0.25	16	32
2003	1	8	16	32	4	8	0.004	0.04	0.12	0.5	16	32
2004	2	32	16	32	8	16	0.004	0.08	0.12	0.25	8	16
2005	1	32	32	64	4	16	0.004	0.008	0.12	0.25	16	32
2006	2	32	32	64	4	16	0.004	0.004	0.12	0.5	32	64

AZM=Azithromycin;

CEFT=Ceftriaxone;

CIP=Ciprofloxacin;

PEN=Penicillin;

SPT=Spectinomycin;

TET=Tetracycline

**Fig. 2.**
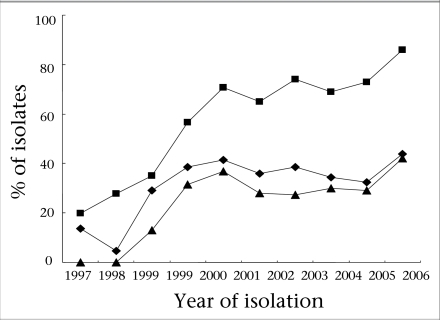
Prevalence of PPNG ♦, TRNG ▪ and both PPNG and TRNG ▴ *N. gonorrhoeae* isolates during 1997-2006 interval)

The presence of PPNG and TRNG among the isolates was determined by PCR. Approximately 14% of the isolates were PPNG in 1997 compared to 44% in 2006. Of the isolates from 1997, 20% were TRNG compared to 86% in 2006. None of the isolates was both PPNG and TRNG in 1997 and 1998 compared to 42% in 2006 (Fig. 2).

The presence and absence of the plasmids were determined by PCR amplification and detection of amplicon by agarose gel electrophoresis and ethidium bromide-staining; PCR=Polymerase chain reaction; PPNG=Penicillinase-producing *N. gonorrhoeae*; TRNG=Tetracycline-resistant *N. gonorrhoeae*

More than 90% of the isolates from 1997 to 2006 carried 3.2-MDa African type PPNG plasmid. However, 4.5% (8/175) and 4.5% (5/131) of the PPNG isolates of 2002 and 2003 respectively carried a Toronto-type plasmid respectively. Of the TRNG isolates, 11% (2/18), 2% (1/49), 7.5% (9/119), 3.2% (6/186), and 2% (5/252) in 1998, 1999, 2000, 2002, and 2003 carried 1600-bp American type *tet*M gene in the 25.2-MDa conjugative plasmid respectively, and the remaining TRNG isolates carried 700 Dutch type *tet*M gene in the 25.2-MDa conjugative plasmid (data not shown).

The prevalence of isolates resistant to three or more antimicrobial agents during 1997-2006 was further determined. Six percent, 27%, 16%, 43%, 50%, 40%, 42%, 51%, 47%, and 44% of the isolates in 1997, 1998, 1999, 2000, 2001, 2002, 2003, 2004, 2005, and 2006 respectively were resistant to three or more antimicrobial agents. Of the multidrug-resistant isolates, none was both PPNG and TRNG in 1997 and 1998, and 33% (10/21), 62% (66/92), 61% (54/74), 66% (128/193), 64% (93/145), 59% (27/46), 63% (32/53), and 83% (24/29) of the isolates from 1999, 2000, 2001, 2002, 2003, 2004, 2005, and 2006 were both PPNG and TRNG respectively. One multidrug-resistant isolate from 2002 was both PPNG and TRNG and was resistant to ciprofloxacin and azithromycin. Similarly, one multidrug-resistant isolate from 2003 and one from 2005 were both PPNG and TRNG and were also resistant to ciprofloxacin and spectinomycin.

## DISCUSSION

The control of gonococcal infection is important considering the high incidence of acute infections, complications, and sequelae and its role in facilitating acquisition and transmission of HIV (17,18). The knowledge of antimicrobial susceptibility of *N. gonorrhoeae* is a prerequisite for the proper treatment and control of gonococcal infection. A regional programme for monitoring gonococcal antimicrobial susceptibility has been established in developed countries, such as the USA, Canada, Australia, and The Netherlands. However, in developing countries where the burden of disease is high and the resistance is the greatest, such activity rarely exists (2,19–21). In Bangladesh, there was no established antimicrobial susceptibility surveillance for *N. gonorrhoeae*. In the absence of laboratory data and an established monitoring system, selection of appropriate antimicrobials for the empirical treatment of gonorrhoea is difficult. We initiated the antimicrobial susceptibility surveillance in Dhaka in 1997, and subsequently, the surveillance was extended to three major cities.

We examined 1,767 gonococcal isolates cultured from street-based, brothel-based, hotel-based female sex workers, male STI patients, male having sex with male (MSM) population, male truckers, and male and female STI patients during 1997-2006. Although the incidence of gonococcal infection among males and females with high-risk behaviour and from general population in Bangladesh is not known, the isolates could be reasonably considered to be representative of gonococcal strains in the country based on the fact that the isolates tested in the study were cultured from different high-risk population and from different parts of the country.

A rapid increase in resistance to ciprofloxacin was observed during the study. Such increase in resistance has been documented in some Western pacific countries, such as in China (85.2%), Hong Kong (79.5%, The Philippines (37.9%), and Viet Nam (42.7%) (2). Ciprofloxacin has been extensively used in Bangladesh for the treatment of suspected gonorrhoea as it is relatively cheap and effective, and only a single oral dose is required. As a consequence of the long-time and large-scale use of this group of antimicrobial agents in areas where over-the-counter availability of drugs without prescription is common, a substantial increase in resistant strains may occur (22). Data from the present study seem to reflect the consequence of the long-standing usage of ciprofloxacin for the treatment of suspected gonorrhoea using syndromic management at STI clinics in Bangladesh.

Resistance to penicillin and tetracycline may be either chromosomally-mediated or plasmidmediated. Chromosomally-mediated resistance to penicillin is of low-level resistance and results from additive effect of mutations at multiple loci, including penA, mtr, and penB while plasmid-mediated resistance is due to PPNG encoding a TEM-1 type β-lactamase. Approximately half of the isolates during the study were resistant to penicillin. Of the penicillin-resistant isolates, the prevalence of PPNG increased from 28% to 88% during 1997-2006. Results of plasmid analysis showed that most isolates carried an African type plasmid; however, isolates carrying Toronto-type plasmid were introduced in 2002, and there was a steady increase since then. All the isolates carrying Toronto-type PPNG plasmid during 2002 were isolated from long-distance truck-divers, and in 2003, isolates carrying Toronto-type PPNG plasmid were isolated from sex workers in Dhaka. This might be due to acquisition of *N. gonorrhoeae* clone with Toronto-type PPNG plasmid by long-distance truckers and subsequent transmission to sex workers.

Although more than three-fourths of the isolates were resistant to tetracycline, it is not currently used for the treatment of gonorrhoea in Bangladesh. This might be due to the fact that tetracycline is used for many other illnesses, and doxycycline is still used for the treatment of *Chlamydia*-associated infection in Bangladesh. High-level plasmid-mediated resistance to tetracycline is due to the acquisition of *tet*M gene by the conjugative plasmid of *N. gonorrhoeae*, resulting in a 25.2-MDa plasmid. Tetracycline-resistant *N. gonorrhoeae* isolates are likely to spread more quickly than PPNG isolates because of the presence of *tet*M plasmid in other flora found in the genital tract that may act as a reservoir. Of the tetracycline-resistant isolates, the prevalence of TRNG increased from 30% to 95% during 1997-2006. The increase in the prevalence of both PPNG and TRNG isolates might be due to acquisition of penicillinase plasmid in the TRNG isolates as transfer of penicillinase plasmid requires the presence of conjugative plasmid (23).

The most striking finding of the present study is the emergence of isolates resistant to three or more antimicrobial agents. More than half of the isolates are resistant to three drugs, including ciprofloxacin, the first-line therapy for gonorrhoea. Of the multidrug-resistant isolates, more then half were both PPNG and TRNG. Besides this, isolates resistant to four antimicrobial agents have also been identified.

Antimicrobial susceptibility data play a major role in updating or revising the national guideline for the management of STIs. Based on our surveillance data, the National AIDS and STD Programme, Ministry of Health and Family Welfare, Government of Bangladesh, has revised the national guidelines for the management of STIs in 2007 and recommended cefixime as the first-line therapy for gonorrhoea. The current alternatives to quinolones for gonococcal infection are the extended spectrum or third-generation cephalosporins. All the isolates in the present study were susceptible to ceftriaxone and cefixime.

Considering the rapidly-changing pattern of gonococcal antimicrobial susceptibility, it is important to maintain the antimicrobial susceptibility-monitoring programme, periodical analysis of susceptibility data, and updating the treatment guidelines for successful STI/HIV intervention programmes.

## ACKNOWLEDGEMENTS

This study was conducted at ICDDR,B with the support of Cooperative Agreement No. HRN-A-00-96-90005-00 from the United States Agency for International Development (USAID). ICDDR,B acknowledges with gratitude the commitment of USAID to the Centre's research efforts.
